# Characterization of Specific Immune Responses to Different *Aspergillus A*ntigens during the Course of Invasive Aspergillosis in Hematologic Patients

**DOI:** 10.1371/journal.pone.0074326

**Published:** 2013-09-04

**Authors:** Leonardo Potenza, Daniela Vallerini, Patrizia Barozzi, Giovanni Riva, Fabio Forghieri, Anne Beauvais, Remi Beau, Anna Candoni, Johan Maertens, Giulio Rossi, Monica Morselli, Eleonora Zanetti, Chiara Quadrelli, Mauro Codeluppi, Giovanni Guaraldi, Livio Pagano, Morena Caira, Cinzia Del Giovane, Monica Maccaferri, Alessandro Stefani, Uliano Morandi, Giovanni Tazzioli, Massimo Girardis, Mario Delia, Giorgina Specchia, Giuseppe Longo, Roberto Marasca, Franco Narni, Francesco Merli, Annalisa Imovilli, Giovanni Apolone, Agostinho Carvalho, Patrizia Comoli, Luigina Romani, Jean Paul Latgè, Mario Luppi

**Affiliations:** 1 Section of Hematology, Department of Surgical and Medical Sciences, University of Modena and Reggio Emilia, Azienda Ospedaliera Policlinico, Modena, Italy; 2 Unitè des Aspergillus, Pasteur Institut, Paris, France; 3 Hematology and Bone Marrow Transplantation, Udine, Italy; 4 Department of Hematology, Universitaire Ziekenhuizen Leuven, Campus Gasthuisberg, Leuven, Belgium; 5 Section of Histopathology, IRCCS/Arcispedale S.Maria Nuova Reggio, Emilia, Italy; 7 Department of Hematology, Università Cattolica del Sacro Cuore, Rome, Italy; 6 Infectious Diseases Clinics, Department of Surgical and Medical Sciences, University of Modena and Reggio Emilia, Azienda Ospedaliera Policlinico, Modena, Italy; 8 Division of Thoracic Surgery, Department of Surgical and Medical Sciences, University of Modena and Reggio Emilia, Azienda Ospedaliera Policlinico, Modena, Italy; 9 Division of General Surgery, Department of Surgical and Medical Sciences, University of Modena and Reggio Emilia, Azienda Ospedaliera Policlinico, Modena, Italy; 10 Division of Anaesthesiology and Intensive Care, University of Modena and Reggio Emilia, Azienda Ospedaliera Policlinico, Modena, Italy; 11 Hematology Department, DAP, University of Bari, Bari, Italy; 12 Division of Hematology, IRCCS/Arcispedale S.Maria Nuova Reggio, Emilia, Italy; 13 Department of Experimental Medicine and Biochemical Sciences, University of Perugia, Perugia, Italy; 14 Pediatric Hematology/Oncology and Transplantation, IRCCS S. Matteo Hospital, Pavia, Italy; University of Medicine and Dentistry of New Jersey - New Jersey Medical School, United States of America

## Abstract

Several studies in mouse model of invasive aspergillosis (IA) and in healthy donors have shown that different *Aspergillus* antigens may stimulate different adaptive immune responses. However, the occurrence of *Aspergillus*-specific T cells have not yet been reported in patients with the disease. In patients with IA, we have investigated during the infection: a) whether and how specific T-cell responses to different *Aspergillus* antigens occur and develop; b) which antigens elicit the highest frequencies of protective immune responses and, c) whether such protective T cells could be expanded ex-vivo. Forty hematologic patients have been studied, including 22 patients with IA and 18 controls. Specific T cells producing IL-10, IFN-γ, IL-4 and IL-17A have been characterized through enzyme linked immunospot and cytokine secretion assays on 88 peripheral blood (PB) samples, by using the following recombinant antigens: GEL1p, CRF1p, PEP1p, SOD1p, α1–3glucan, β1–3glucan, galactomannan. Specific T cells were expanded through short term culture. *Aspergillus*-specific T cells producing non-protective interleukin-10 (IL-10) and protective interferon-gamma (IFN-γ) have been detected to all the antigens only in IA patients. Lower numbers of specific T cells producing IL-4 and IL-17A have also been shown. Protective T cells targeted predominantly *Aspergillus* cell wall antigens, tended to increase during the IA course and to be associated with a better clinical outcome. *Aspergillus*-specific T cells could be successfully generated from the PB of 8 out of 8 patients with IA and included cytotoxic subsets able to lyse *Aspergillus* hyphae. *Aspergillus* specific T-cell responses contribute to the clearance of the pathogen in immunosuppressed patients with IA and *Aspergillus* cell wall antigens are those mainly targeted by protective immune responses. Cytotoxic specific T cells can be expanded from immunosuppressed patients even during the infection by using the above mentioned antigens. These findings may be exploited for immunotherapeutic purposes in patients with IA.

## Introduction

In mouse model of invasive aspergillosis (IA), previous studies have reported that adaptive immunity contribute to the defence of the host against fungi of the genus *Aspergillus* and that *Aspergillus*-specific T cells producing interferon-gamma (IFN-γ) may result protective, while those producing interleukin-10 (IL-10) may result non-protective to the fungus [1]–[3]. In healthy subjects, it has subsequently been demonstrated that T cells may proliferate and produce different amount of IL-10, IFN-γ, IL-4 and IL-17A in response to *Aspergillus* recombinant antigens and that the protective T cells may be expanded from their peripheral blood as possible source of adoptive therapy [4], [5].

However, the emergence and changes of dynamics of *Aspergillus*-specific T cells have not yet been described in patients with IA and the above mentioned results could be barely applicable to immunosuppressed patients during the common clinical practice.

Thus, we have phenotypically and functionally characterized *Aspergillus-*specific T-cell responses against seven different *Aspergillus* recombinant antigens either in patients with IA, during the course of the infection, or in a comparable number of controls, and identified which antigens are most frequently targeted by protective immune responses**.** The results in patients with IA and controls have been compared with those obtained in healthy subjects (HS). Furthermore, *Aspergillus-*specific T cells have been expanded from the peripheral blood of patients with IA.

## Materials and Methods

### Ethics Statement

Written informed consent was obtained according to the Declaration of Helsinki, and after the local Ethical Committee’s study approval (Comitato Etico Provinciale di Modena – protocol n° 2414–63/11).

### Patients

Forty hematologic patients were studied: 22 patients with IA, 16 proven and 6 probable cases according to the current diagnostic criteria [6], and 18 patients with infectious complications other than IA or without infections (Table 1). Furthermore to validate the results in IA and control patients we have also studied the occurrence of specific immune response in 13 HS.

**Table 1:** Clinical characteristics of the patients.

Patients n°	Sex/Age (yr)	Underlying disorder or conditions	Site of Infection	Isolates/biopsy	GM	ELISpot samples/n° positives	Antifungal Treatment
1	M/50	AML	Lung	IA	pos	3/3	L-AmB
2	M/62	MM	Lung	IA	pos	3/3	Voriconazole
3	M/64	AML	Lung	IA	neg	2/1	Caspofungin, L-AmB
4	F/55	AML	Lung	IA	neg	2/2	L-AmB, Voriconazole
5	M/52	AML	Lung	IA	pos	2/2	Caspofungin
6	M/49	ALL	Lung/Visceral	IA	pos	4/4	L-AmB, Caspofungin
7	M/18	AML	Visceral	IA	pos	4/4	L-AmB, Caspofungin, Voriconazole
8	F/45	AML	Lung	IA	neg	3/3	Voriconazole
9	M/22	ALL	Lung	IA	neg	3/3	L-Amb, Caspofungin, Voriconazole
10	M/69	AML	Lung	IA	neg	2/2	L-AmB
11	F/44	AML	Lung	Aspergillus spp/probable	pos	3/3	L-AmB
12	F/19	AML	Lung	IA	neg	3/3	Caspofungin, Voriconazole
13	M/71	CLL	Lung	IA	neg	1/1	Voriconazole
14	M/22	AML	Lung	Aspergillus fumigatus/probable	pos	3/3	L-AmB
15	M/55	DLCBL	Lung	IA	neg	2/2	Voriconazole
16	F/48	AML	Lung	−/probable	pos	1/1	Caspofungin
17	F/40	AML	Lung	−/probable	pos	5/5	L-AmB
18	F/58	AML	Lung	IA	pos	2/2	L-AmB
19	M/51	AML	Lung	−/probable	pos	2/1	Caspofungin
20	F/47	AML	Lung	Aspergillus fumigatus/probable	pos	1/1	Voriconazole
21	M/46	AlloSCT	CNS/Lung	IA	pos	1/1	Voriconazole
22	M/43	AlloSCT	Lung	IA	pos	2/2	L-AmB
23	M/59	AML	Lung	RSV	neg	2/0	Posoconazole
24	F/23	ALL	–	–	–	3/0	L-AmB
25	M/58	AlloSCT	Lung	M. Tuberculosis	neg	3/0	Posoconazole
26	M/53	AML	Lung	Parainfluenza Virus	neg	2/0	Posoconazole
27	M/79	AML	Lung	Pseudomonas aeruginosa	neg	1/0	Posocanazole
28	M/68	ALL	–	–	–	2/0	–
29	M/62	AlloSCT	Lung	M. Tuberculosis	neg	1/0	–
30	F/72	AutoSCT	Typhlitis	Pseudomonas aeruginosa	neg	1/0	–
31	F/69	AML	Lung	CMV	neg	1/0	Posaconazole
32	M/55	NHL-T	Lung	CMV	neg	2/0	L-AmB, Voriconazole
33	F/68	DLBCL	Lung	Solid cancer	neg	3/0	–
34	M/73	ALL	Sepsis	E. choli	neg	2/0	Caspofungin
35	M/71	AML	Lung	Stenotrophomonas maltophilia	neg	3/0	L-AmB
36	F/63	MDS	Lung	Acinetobacter baumannii	neg	2/0	L-AmB
37	F/59	AML	–	–	–	2/0	–
38	F/69	ALL	Sepsis	Enterococcus faecium	neg	1/0	Caspofungin
39	M/68	AML	Lung	Enterococcus faecalis	neg	1/0	L-AmB
40	F/65	AML	Lung	Stenotrophomonas maltophilia	neg	2/0	L-AmB

AML = acute myeloid leukemia; ALL = acute lymphoblastic leukemia; CLL = chronic lymphocytic leukemia; AlloSCT = allogeneic stem cell transplant; AutoSCT = autologous stem cell transplant; DLCBL = diffuse large B cell lymphoma; NHL = non Hodgkin lymphoma; MDS = myelodysplastic syndrome; CNS = central nervous system; IA = proven Invasive Aspergillosis; RSV = respiratory syncytial virus; CMV = cytomegalovirus; E. coli = Escherichia coli; pos = positive; neg = negative; L-AmB = liposomal amphotericin B.

### ELISpot for the Detection of *Aspergillus*-specific T Cells

The enzyme linked immunospot (ELISpot) assay has been performed to detect *Aspergillus*-specific T cell producing IL-10, IFN-γ, IL-4 and IL-17A, on 101 peripheral blood samples, by using seven recombinant antigens of *Aspergillus*. Peripheral blood mononuclear cells (PBMCs) were obtained by Ficoll-Hypaque gradient centrifugation and co-cultured for 18–40 hours on a 96-well bottom plates coated with anti-cytokine antibodies, namely anti-IL10, anti-IFN-γ, anti-IL4 and anti-IL17A, as previously reported [7], [8]. The viability of the analysed T cells has been determined by stimulation with anti-CD3 antibody. *Aspergillus*-specific T cells producing IL-10, IFN-γ, IL-4 were evaluated in 13 out of 13 HS; while those producing IL-17A in 4 out of 13 HS.

Based on our previous studies, a sample was considered positive if all the followings were fulfilled: 1) the presence of at least 10 spot forming cells (SFCs); 2) the presence of more than 10 SFCs in the sample compared with the negative control; 3) the presence of a stimulation index of ≥20 (defined as the ratio of the number of SFCs in the positive control to that in the negative control) [7]–[9].

The 7 different recombinant antigens of *Aspergillus* were used at a concentration of 5–7 µg/mL, and included: 1) two glycosylphosphatidylinositol (GPI)-anchored proteins namely 1,3-β glucanosyltransferase (GEL1p) and an ortholog of Crh1p associated in β1,6 glucan-chitin linkages (CRF1p) in S*accharomyces cerevisiae*; 2) two secreted proteins, such as the aspartic protease (PEP1p) and superoxide dismutase (SOD1p); 3) three major polysaccharides of the cell wall such as α1–3 glucan, β1–3 glucan, and galactomannan. All the antigens have been produced, purified and harnessed as previously reported [5], [10], [11].

ELISpot results have been reported as median spot forming cells (SFCs)/10^∧^6 PBMCs with their 25^th^ and 75^th^ percentile values.

### Cytofluorimetric and Functional Characterization of *Aspergillus-*specific T cell

In patients with IA, on peripheral blood samples resulted positive at the ELISpot assay, we have phenotypically and functionally characterized Aspergillus-specific T cells to the seven recombinant antigens by using the cytokine secretion assay (CSA) for IFN-γ, IL-10, IL-4, IL-17A, according to the manufacturer’s instructions (CSA Detection Kit; Miltenyi Biotec), as already reported [7], [8], with few modifications. Briefly, 1×10^6^ PBMCs were stimulated with Aspergillus recombinant antigens, at a final concentrations of 5–7 µg/mL, from 17 to 40 hours, according to the cytokine studied. After stimulation, PBMCs were immunostained with IFN-γ, IL-10, IL-4, or IL-17 catch reagent, and the phenotype of the cytokine producing T cells was directly assessed after sample counterstaining, as reported [7], [8]. Unstimulated and PHA-stimulated PBMCs were used as negative and positive controls, respectively. Cells were acquired on a FACSCalibur flow cytometer (BD Biosciences) and analyzed by the use of CellQuest (BD Biosciences) and Summit softwares (Dako). Frequencies of antigen-reactive effector memory (EM) or central memory (CM) T cells were calculated as mean differences compared with unstimulated controls. CD8^+^ or CD4^+^ T cells were gated on CD3^+^ events after passing through a small lymphocyte gate. The phenotype of the cytokine producing cells was directly assessed after sample counterstaining with CD3 APC, CD8 FITC or PerCP, CD4 PerCP or APC, CD62L or CCR7 PE, allowing the identification of EM T cells (CD3+, CD8+ or CD4+, CD62L−/CCR7−) or CM T cells (CD3+, CD8+ or CD4+, CD62L+/CCR7+). Results were expressed as mean percentages of either PB CD8^+^ T cells or CD4^+^ T cells ± standard deviation (SD).

### *Aspergillus*-specific T cells Expansion and Demonstration of Lytic Activity

*Aspergillus*-specific T cells were obtained by culturing PBMCs from 8 patients with IA: 1) alone, as control; 2) with a mixture of *Aspergillus* recombinant antigens (PEP1p, GEL1p, α1–3 glucan and β1–3 glucan) (8 ug/mL); and 3) with heat killed germinated conidia (100.000/ml), for 13 days in the presence of IL-2, IL-7 and IL-5. To further functionally characterize *Aspergillus-*specific T cells, we investigated the presence of specific-cytotoxic T cell subsets by means of the colorimetric assay with (2,3-bis[2-methoxy-4-nitro-5-sulfophenyl]2H-tetrazolium-5-carboxyanilide) sodium salt (XTT; Sigma) plus coenzyme Q0 (2,3-dimethoxy-5-methyl-1,4-benzoquinone; Sigma). Before the assessment of their lytic activity, cells from short term cultures were phenotypically characterized. An *Aspergillus fumigatus* isolate from one of the patients has been used as target. T-cell induced specific hyphal damage has been assessed as follows. Briefly, conidia of *Aspergillus fumigatus* (1.5×10^4^ per well) were plated in a 96-well flat bottom plate and incubated at 37°C for 16 hours to allow germination. On the following day, unstimulated and stimulated anti-*Aspergillus* T cells were added at an effector-to-target (E/T) ratio of 3∶1 and 5∶1 and incubated at 37°C with 5% CO_2_ for 2 and 22 hours, respectively, and each experimental condition was performed in triplicate. Anti-hyphal activity was expressed as a specific hyphal damage and was calculated according to the formula: percent hyphal damage = [1−((X−Y)/C)] ×100, where X is the absorbance of experimental wells with stimulated cells, Y is the absorbance of experimental wells with unstimulated cells and C is the absorbance of control wells with hyphae only. In three out of 8 patients, to evaluate whether anti-hyphal activity of *Aspergillus*-specific T cells was similar to those of antigen presenting cells (APCs) and polymorphonuclears (PMNs) and how it was accomplished, we compared the killing ability of anti-*Aspergillus* T cells, APCs, PMNs and the supernatant of T-cell cultures. Furthermore, we quantified the amount of cytokines (Granzyme B and IFN-γ) produced by PBMCs after 24 h culture with the above mentioned antigens by enzyme linked immunosorbent assay (ELISA).

### Phases of Invasive *Aspergillosis*

Based on the kinetics of radiologic signs of pulmonary IA on HRCT [12], [13], the infection course has been divided into four phases, defined from t1 to t4, and corresponding to the number of days elapsed from the radiological diagnosis of IA (t1, from the radiologic disclosure of the infectious lesions until day +14; t2, from day +15 until day +30; t3, from day +31 to day +45; t4, from day +46 to day 60 days from the radiological diagnosis of IA). Only two patients had a sample collected more than 61 days after the radiological demonstration of IA and the respective results were shown as t4+.

### Statistical Analysis

The Fischer’s exact test has been used to determine if there were nonrandom associations between two categorical variables and the outcome of the patients; the association between the number of antigens targeted by specific T cells and the status of the subjects (HS or patients with IA). Chi-squared test has been used to compare the rate of samples with more than 100 SFCs/10^∧^6 PBMCs to all the antigens between HS and patients with IA. The paired T test has been used to compare the rates of fungal hyphae lysis between the single cell fractions and their associations. *P* values below.05 were considered significant. The results were obtained using the Stata Software (11.0, College Station, Texas, USA).

## Results

### Identification of *Aspergillus*-specific T cells

**Figure 1:**
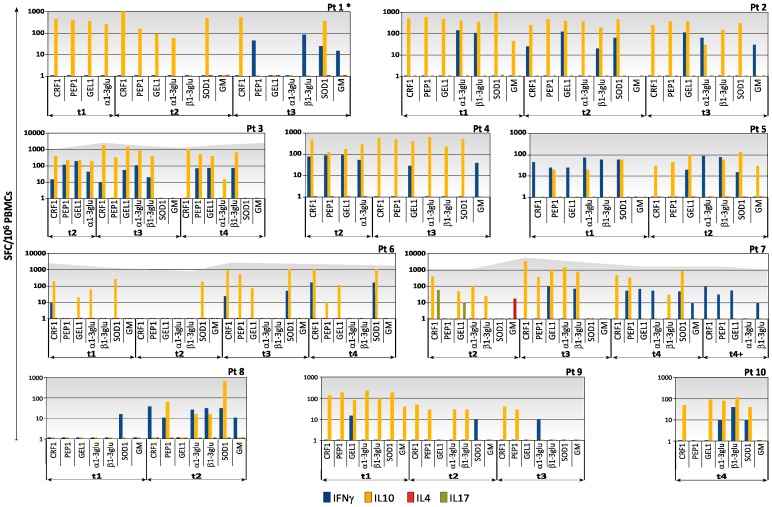
Kinetics of specific T-cell responses to the seven recombinant antigens of *Aspergillus* by IFN-γ, IL-10, and IL-4 ELISpot assay in 22 patients with invasive aspergillosis (IA), patient 1 to patient 10.

**Figure 2:**
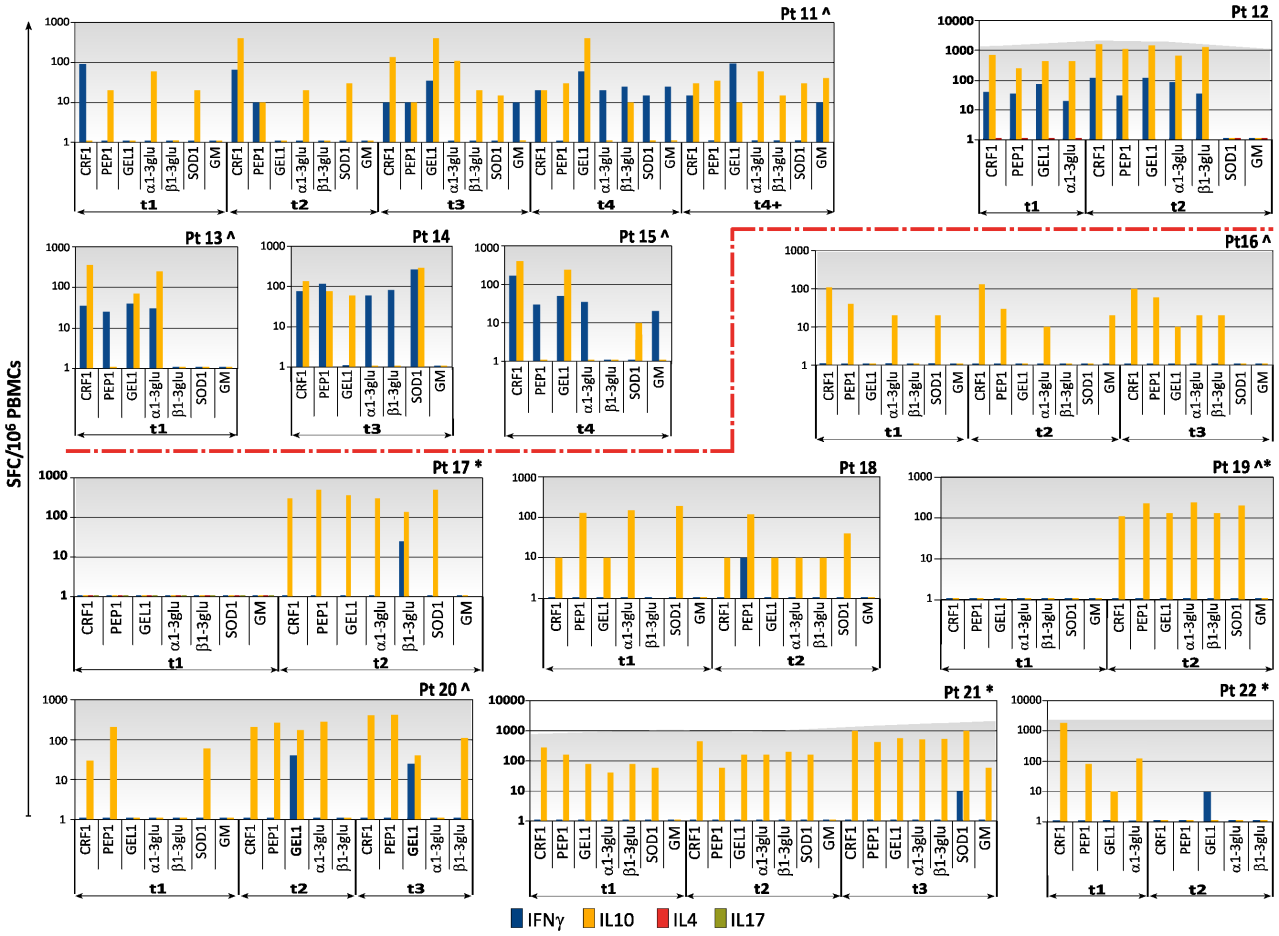
Kinetics of specific T-cell responses to the seven recombinant antigens of *Aspergillus* by IFN-γ, IL-10, and IL-4 ELISpot assay in 22 patients with invasive aspergillosis (IA), patient 11 to patient 22. Blue columns represent the number of *Aspergillus*-specific T cells producing IFN-γ; yellow columns represent the number of *Aspergillus*-specific T cells producing IL-10; green columns represent the number of *Aspergillus*-specific T cells producing IL-17A; red columns represent the number of *Aspergillus*-specific T cells producing IL-4; dark gray background represents T-cell responses in wells with anti-CD3 antibody. Vertical axis shows the number of spot-forming cells (SFCs) per million peripheral blood mononuclear cells (PBMCs); horizontal axis indicates the antigen, which the specific immune responses are directed to and the phase of IA calculated as the number of days elapsed from the radiological diagnosis of the infection (t1, from the radiologic disclosure of the infectious lesions until day +14; t2, from day +15 until day +30; t3, from day +31 to day +45; t4, from day +46 to day 60 days; t4+ >61 days). CRF1 =  ortholog of Crh1p associated in β1,6 glucan-chitin linkages; PEP1 =  aspartic protease; GEL1 = 1,3-β glucanosyltransferase; α1–3 glu = α1–3 glucan; β1–3 glu = β1–3 glucan; SOD1 =  superoxide dismutase; and GM = galactomannan. Pt = patient. * = patients with unfavorable outcome. ^∧^ = patients with probable IA. The red dotted line divides the two groups of patients: from pt n° 1 to pt n° 15 with *Aspergillus*-specific T cells producing IFN-γ to two or more antigens; from pt n° 16 to pt n° 22 without *Aspergillus*-specific T cells producing IFN-γ or with such cells to only one antigen.

A median of 2 time-points were analysed for each patient (range from 1 to 5) (Table 1). All the patients with IA presented *Aspergillus*-specific T cells at least at one time point (Figures 1 and 2).

**Figure 3:**
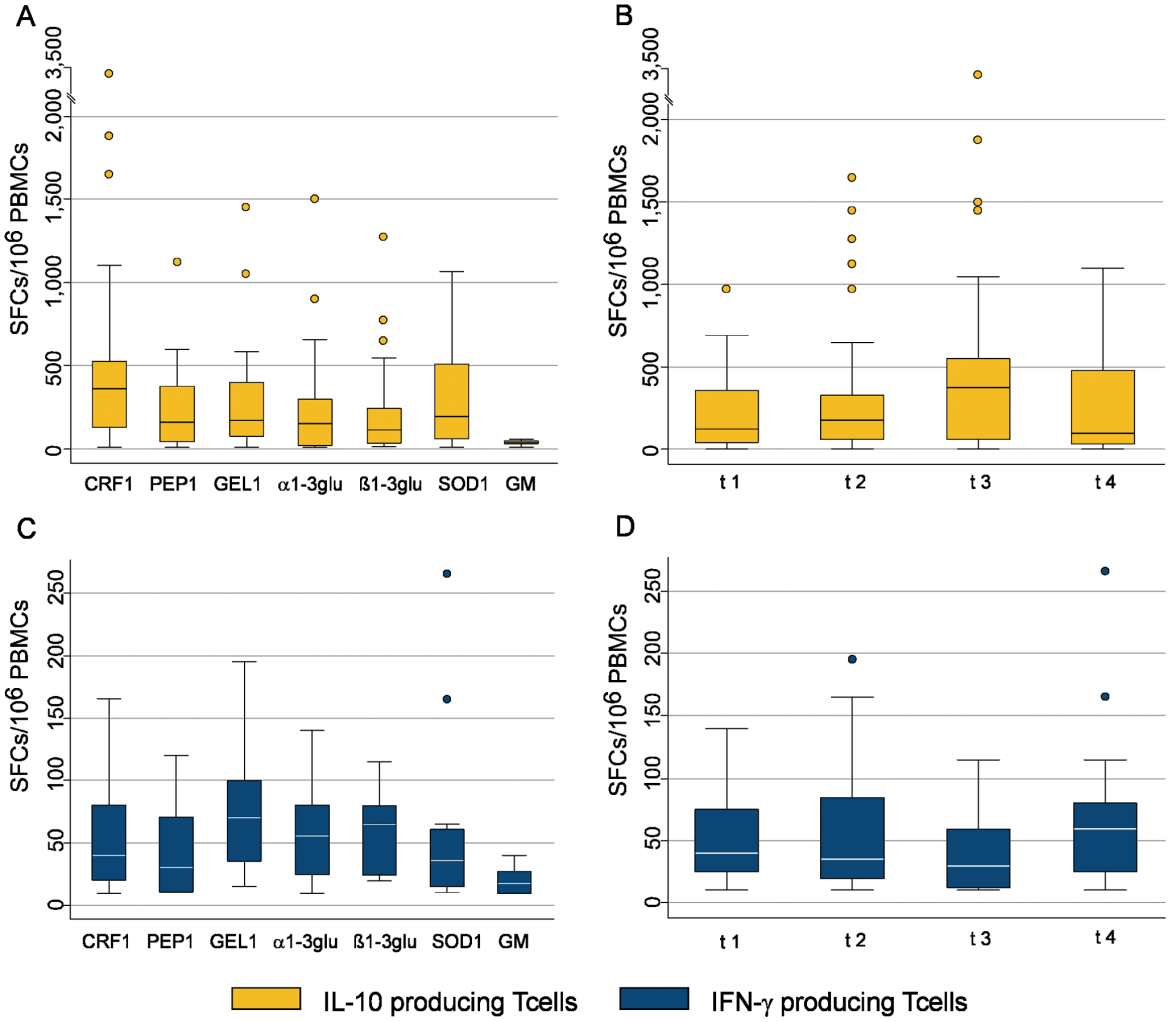
*Aspergillus*-specific T-cell responses to the 7 recombinant antigens during the course of Invasive Aspegillosis (IA). **A,B,C,D. A.** Box plots showing specific immune responses producing IL-10 (yellow columns) to any single recombinant antigen. The horizontal axis represents the antigen, which the specific immune responses are directed to. **B.** Box plot showing specific immune responses producing IL-10 (yellow columns) against all the 7 recombinant antigens at the four phases of the IA. The horizontal axis represents the different phases of IA. **C.** Box plot showing specific immune responses producing IFN-γ (blue columns) to any single recombinant antigen. The horizontal axis represents the antigen, which the specific immune responses are directed to. **D.** Box plot showing specific immune responses producing IFN-γ (blue columns) against all the 7 recombinant antigens at the four phases of the IA. The horizontal axis represents the different phases of IA. The vertical axis shows the number of spot-forming cells (SFCs) per million peripheral blood mononuclear cells (PBMCs).The upper horizontal line represents the upper adjacent value. The upper hinge of the boxes represents the 75^th^ percentile. The middle horizontal line of the boxes represents the median value. The lower hinge of the boxes represents the 25^th^ percentile. The lower horizontal line represents the lower adjacent value. Yellow dots and blue dots are outrange values.

Specific T cells (median SFCs/10^∧^6 PBMCs with their 25^th^ and 75^th^ percentile values) producing IL-10 were detected to all the antigens: CRF1p 375 (130–550); PEP1p 160 (45–375); GEL1p 175 (75–400); α1–3glucan 150 (20–300); β1–3glucan 110 (30–240); SOD1p 198 (60–510); Galactomannan 40 (30–45). The frequencies of specific T cells producing IL-10 to all the antigens increased in the first three phases [125 (42.5–360) at t1; 175 (60–330) at t2; 375 (60–580) at t3] and tended to decrease in the fourth phase of the infection [97.5 (30–480) at t4] (Figure 3 A,B).

Specific T cells producing IFN-γ (median SFCs/10^∧^6 PBMCs with their 25^th^ and 75^th^ percentile values) were also detected to all the antigens: CRF1p 40 (20–80); PEP1p 30 (10–70); GEL1p 70 (35–100); α1–3glucan 55 (25–80); β1–3glucan 65 (25–80); SOD1p 35 (15–60); Galactomannan 17.5 (10–27.5). The frequencies of specific T cells producing IFN-γ to all the antigens remained stable in the first three phases (40 (25–75) at t1; 35 (20–85) at t2; 30 (12.5–60) at t3) and increased after 45 days of IA [60 (25–80) at t4] (Figure 3 C,D).

Patients who never showed specific T cells producing IFN-γ or demonstrated such cells against only one antigen (pts 16, 17, 18, 19, 20, 21, 22), presented more frequently an unfavourable outcome compared with patients presenting protective immune responses versus two or more antigens. Actually, 4 (pts 17, 19, 21, 22) out of 7 patients died of IA in the first group, while only 1 (pt n°1) out of 15 patients died of the infection in the second group (*p* = 0.021) (Figures 1 and 2).

Specific T cells producing IL-4 and IL-17A could be detected in only one patient (pt7; Figure 1) by the ELISpot assay.

In the 18 control patients the occurrence of *Aspergillus*-specific T cells could not be demonstrated (Table 1).

**Figure 4:**
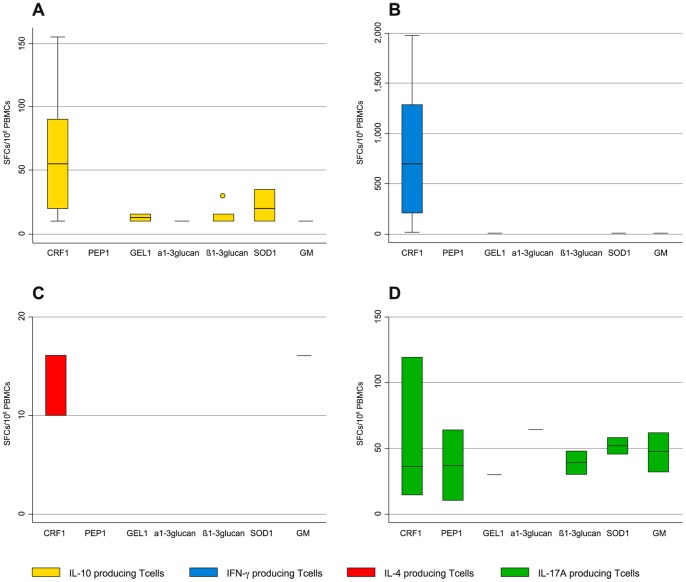
*Aspergillus*-specific T-cell responses to the 7 recombinant antigens in 13 Healthy Subjects. **A,B,C,D. A.** **** Box plots showing specific immune responses producing IFN-γ (blue columns) to any single recombinant antigen. **B.** Box plot showing specific immune responses producing IL-10 (yellow columns) to any single recombinant antigen **C.** Box plot showing specific immune responses producing IL-4 (red columns) to any single recombinant antigen. **D.** Box plot showing specific immune responses producing IL-17A (green columns) to any single recombinant antigen. The vertical axis shows the number of spot-forming cells (SFCs) per million peripheral blood mononuclear cells (PBMCs). The horizontal axis represents the antigens, which the specific immune responses are directed to. The upper horizontal line represents the upper adjacent value. The upper hinge of the boxes represents the 75^th^ percentile. The middle horizontal line of the boxes represents the median value. The lower hinge of the boxes represents the 25^th^ percentile. The lower horizontal line represents the lower adjacent value. Dots are outrange values.

In the HS, specific T cells (median SFCs/10^∧^6 PBMCs with their 25^th^ and 75^th^ percentile values) producing IL-10 were detected to CRF1p 55 (20–90) in 11 out of 13; GEL1p 12.5 (10–15) in 2 out of 13; α1–3glucan 10 (10–10) in 1 out of 13; β1–3glucan 10 (10–15) in 6 out of 13; SOD1p 20 (10–35) in 3 out of 13; Galactomannan 10 (10–15) in 1 out of 13. No HS demonstrated specific T cells producing IL-10 to PEP1p (Figure 4 A).

Specific T cells (median SFCs/10^∧^6 PBMCs with their 25^th^ and 75^th^ percentile values) producing IFN-γ were detected to CRF1p 690 (209–1285) in 10 out of 13; GEL1p 10 (10–10) in 1 out of 13; SOD1p 10 (10–10) in 1 out of 13; Galactomannan 14 (14–14) in 1 out of 13 (Figure 4 B).

Specific T cells (median SFCs/10^∧^6 PBMCs with their 25^th^ and 75^th^ percentile values) producing IL-4 were detected to CRF1p 16 (10–16) in 3 out of 13 and to Galactomannan 16 (16–16) in 1 out of 13 (Figure 4 C).

Specific T cells (median SFCs/10^∧^6 PBMCs with their 25^th^ and 75^th^ percentile values) producing IL-17A were detected to CRF1p 36 (14–120) in 3 out of 4; PEP1 37 (10–64) in 2 out of 4; GEL1p 30 (30–30) in 1 out of 4; α1–3glucan 64 (64–64) in 1 out of 4; β1–3glucan 39 (30–48) in 2 out of 4; SOD1p 52 (46–58) in 2 out of 4; Galactomannan 47 (32–62) in 2 out of 4 (Figure 4 D).

The comparison of specific immune responses producing IL-10 and IFN-γ between patients with IA and HS demonstrated that: 1) patients with IA presented higher number of *Aspergillus*-specific T cells producing IL-10 (100 or more SFCs/10^∧^6 PBMCs) to each of the seven recombinant antigens than HS (55.56% vs 2.2% of the tested samples, *p* = .0001); 2) patients with IA presented wider antigenic specificity (directed to 4 or more antigens) of *Aspergillus*-specific T cells producing either IL-10 (77.27% of the IA patients vs 15.38% of HS, *p* = .0001) or IFN-γ (54.25% of the IA patients vs 0% of HS, *p* = .031).

### Phenotypic and Functional Characterization of *Aspergillus*-specific T cells

**Figure 5:**
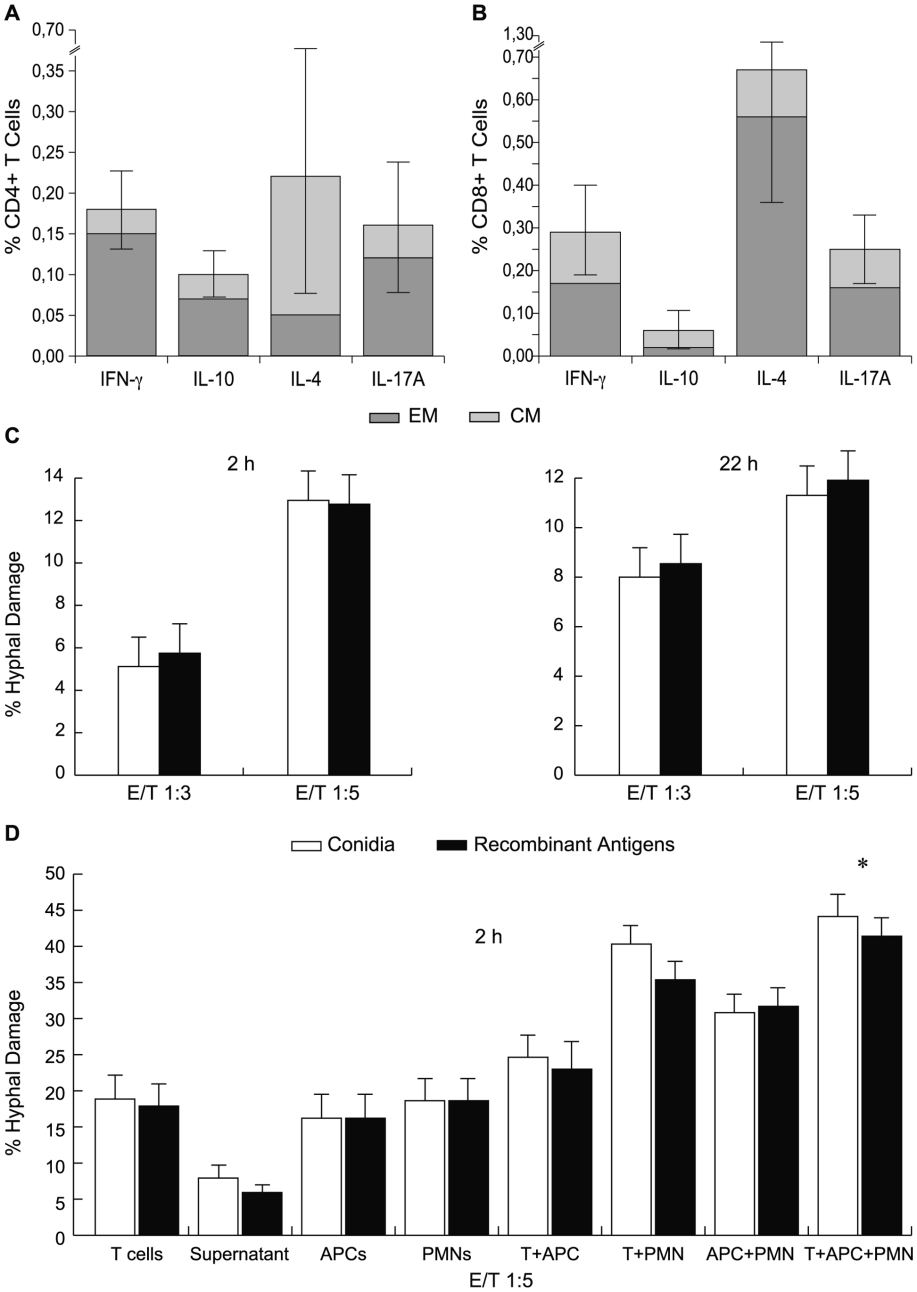
Cytokine production profile and lytic activities of *Aspergillus-*specific T cells. **A, B.** The frequencies of *Aspergillus* specific T cells producing IFNγ, IL-10, IL-4 or IL-17A against the seven recombinant antigens, either as EM (dark gray portion of the columns) or CM (light gray portion of the columns), are shown as mean % positive cells ± standard deviation, computed over the 22 patients with IA. Results are expressed as percentages of either CD4^+^ T cells (A) or of CD8^+^ T cells (B). **C.** White columns represent rates of hyphal damage by *Aspergillus*-specific T cells expanded from PBMCs stimulated with heat killed *Aspergillus* conidia. Black columns represent rates of hyphal damage by *Aspergillus*-specific T cells expanded from PBMCs stimulated with *Aspergillus* recombinant antigens (GEL1, PEP1, α1–3 glucan, β1–3 glucan) at two and twenty two-hour cultures. E/T = effector/target cell ratio. **D.** Rates of hyphal damage by *Aspergillus*-specific T cells expanded with *Aspergillus* either heat killed conidia (white columns) or recombinant antigens (GEL1, PEP1, α1–3 glucan, β1–3 glucan) (black columns); by the supernatant of cultures of *Aspergillus*-specific T cell lines only; by antigen presenting cells (APCs); by polymophonuclears (PMNs) and by combinations of the different three cell fractions from three further patients with IA, at two-hour cultures. * = *P*<.05. E/T = effector/target cell ratio. Results are expressed as mean+−Standard Deviation.

The CSA analysis showed that antigen-specific T cells were: 1) either CD8+ or CD4+ T cells producing IFN-γ (mean ± SD CD8+/CD4+: 0.19±0.13/0.17±0.10%), the former either CM or EM (0,12±0.16/0.17±0.12), the latter predominantly EM (0.15±0.11/0.03±0.03); 2) predominantly CD4+ T cells producing IL-10 (mean CD8+/CD4+: 0.05±0.05/0.10±0.06%) of EM phenotype; 3) predominantly CD8+ T cells producing IL-4 (median CD8+/CD4+: 0.72±0.034/0.22±0.30%) of EM phenotype (mean CD8+ EM/CM 0.56±0.50/0.11±0.15%); 4) either CD8+ or CD4+ T cells producing IL-17A (mean CD8+/CD4+: 0.20±0.11/0.16±0.16%) both mainly of EM phenotype (CD8+ EM/CM = 0.16±0.13/0.09±0.07; CD4+ EM/CM = 0.12±0.16/0.04±0.03) (Figure 5A,B).

### Lytic Activity of *Aspergillus-*specific T cells

After the 13-day cultures, from five out of five patients with IA, with the mixture of PEP1p, GEL1p, α1–3 glucan and β1–3 glucan, we were able to expand a mean of 95.8% CD3+ cells (95% CI 94.7%–97.8%), either CD4+ or CD8+ (mean values 38.5%/56.2%, 95% CI, 29.3%–44.2% and 51.6%–66.5%, respectively), of either CM or EM phenotype (mean 58.3%/78.6%, 95% CI, 44.2%–59.4% and 70.5%–79.2%, respectively). Specific hyphal damage was demonstrated at either 3∶1 or at 5∶1 effector:target cells ratios, at significant levels (mean +−SD 9.63% +− 3.54, ranges from 5.12% to 12.95%, 95%CI, 6.83%–12.43%), comparable to that observed with T cells expanded with germinated conidia (9.45+−3.17, ranges from 5.75% to 12.77%; 95%CI, 6.87%–12.6%; p>.05) (Figure 5C). Moreover, specific T cells obtained from three further patients at the same culture conditions, at 5∶1 effector:target cells ratio, demonstrated specific hyphal damage comparable to that of either APCs or PMNs (mean lytic rates +−SD, 95% CI: T cells expanded with the mixture of antigens = 17.89+−5.68, 3.78–31.99; APCs = 16.2+−5.84, 1.69–30.70; PMNs = 18.63+−5.60, 4.72–32.54). Only the association of all the cell fractions produced a statistical significant increase of hyphal damage (mean lytic rates +−SD, 95% CI: T cells expanded with the mixture of antigens+APCs+PMNs = 41.4+−4.18, 2.14–9.75; *p*<.05). Of note, the lysis of *Aspergillus* hyphae by using only the supernatant from anti-*Aspergillus* T-cell lines was less than a half of that obtained by incubating hyphae with the whole cytotoxic T-cell lines (CTLs) cultures (mean lytic rates +− SD, 95% CI: supernatant from CTLs expanded with the mixture of antigens = 5.93+−1.52, 2.14–9.714) (Figure 5D). The ELISA quantification showed higher values of Granzyme B and IFN-γ in the 24 hour supernatants from the cultures either stimulated with conidia or the mixture of antigens, when compared with the supernatant from unstimulated controls. Granzyme B (median cytokine value; range): in culture stimulated with conidia: 145 pg/mL (113–173); in culture stimulated with the mixture of antigens: 196 pg/mL (75–239); in unstimulated control: 45 pg/mL (31–150). IFN-γ (median cytokine value; range): in culture stimulated with conidia: 93 pg/mL (11–527); in culture stimulated with the mixture of antigens: 110 pg/mL (5–707); in unstimulated control: 47 pg/mL (4–77).

## Discussion

Here we report, for the first time, how *Aspergillus-*specific immune responses to different *Aspergillus* antigens may emerge and develop in patients with IA. The absence of such immune responses in patients without IA demonstrates that: a) they are specific to *Aspergillus spp*., b) unrelated to the administered treatments, c) and not in antithesis with previous reports and our data showing *Aspergillus* specific T cells in healthy subjects [4]. Indeed, intensive chemotherapy may cause the reduction of such cells at frequencies below the ELISpot threshold, while the occurrence of IA, with the antigen rechallenge, may increase again specific immune responses. Moreover, compared with the results obtained in HS, patients with IA demonstrated noticeably higher frequencies of *Aspergillus*-specific T cells producing IL-10 and *Aspergillus*-specific T cells producing IFN-γ with wider antigenic specificity, implying that, given such a set of antigens, the occurrence of high number of the former cells and of a wide antigenic specificity of the latter may represent the immunologic signature of IA. These data may also provide the rationale for further studies to use the ELISpot as a novel diagnostic tool for IA. Of note, *Aspergillus*-specific T cells producing IL-17A may be detected more frequently and to more antigens in HS than in patients with IA, suggesting that the loss of such cells may be implicated in the pathogenesis of IA. However, further data are necessary to draw any firm conclusions about this.

Our study shows that although IA is predominantly characterized by the presence of specific T cells producing IL-10, protective immune responses may occur since the onset of and tend to increase during IA. The antigens eliciting the highest frequencies of specific T cells producing IFN-γ are all involved in cell wall biosynthesis of *Aspergillus*, namely GEL1p and both glucans, partially in agreement with the results obtained in mice with IA, where GEL1p, CRF1p and α1–3 glucan resulted the antigens associated with a higher activation of type 1 responses [5]. The recognition of polysaccharides is probably mediated by specific T cells through the class II major histocompatibility complex (MHC) on APCs, as previously reported for other infectious agents showing glycoantigens, such as *Staphylococcus aureus*, *Streptococcus pneumoniae* and *Bacteroides fragilis* [14], [15]. Moreover, in our patients, the association between T cells producing IFN-γ to two or more antigens and a more favorable outcome may suggest that such protective T cells play a role in the resolution of the infection.

The functional and phenotypical characterization shows that *Aspergillus-*specific T cells are either CD4+ or CD8+ T cells, and that T cells producing either IL-4 or IL-17A may be detected to all the antigens, suggesting that they are present at very low frequencies in patients with IA. These findings confirm that CD8+ T cells contribute to the immune response to filamentous fungi [7], [16], and are in line with those reports showing that *Aspergillus fumigatus* is a weak inducer of IL-4 and IL-17A responses [5], [17].

The absence of concordance between the Elispot assay and CSA, in particular about the frequencies of specific T cells producing IL-10, IL-4 and IL-17A is not unexpected and reflects the different intrinsic characteristics of the two assays, being the ELISpot assay more suitable to detect low-level responses and to define such responses as either positive or negative, while the CSA more appropriate for a phenotypic discrimination of responding cells, as already reported [7], [16]–[18].

By expanding previous findings, so far obtained only in healthy donors [19]–[21], we have expanded *Aspergillus*-specific T cells from PB of patients with IA using a mixture of recombinant antigens different from CRF1p, and found that such cells may induce direct lysis of *Aspergillus* hyphae, comparable to those obtained by APCs and PMNs. By showing that the hyphal lysis obtained with the supernatant of anti-*Aspergillus* CTLs is lower than that registered with the whole CTLs culture (anti-*Aspergillus* CTLs+supernatant), and that the production of Granzyme B and IFN-γ was higher in stimulated samples, our data suggest that the activity against *Aspergillus* is mediated directly by T cells, either by secreting cytokines or by cell-cell contact, and are consistent with previous reports [22], [23]. The observation that protective immune responses in HS are almost exclusively elicited by CRF1p, while they are predominantly directed to *Aspergillus* cell wall antigens in patients with IA, should be taken into account when planning immunotherapeutic strategies.

In conclusion, specific immune responses to several Aspergillus antigens may be detected in patients with IA and those producing IFN-γ directly mediate the lysis of *Aspergillus* hyphae, possibly contributing to the clearance of the fungus, being their presence also associated with a more favourable outcome. The identification of the antigens eliciting the strongest protective responses in patients with IA may spur further studies to define the complete repertoire of *Aspergillus* specific immune responses during the course of the infection to design therapeutic strategies of either vaccine or autologous cytotoxic cell infusions.

## References

[1] Romani L (2004) Immunity to fungal infections. Nat Rev Immunol 4: 1–23. 14661066 10.1038/nri1255

[2] Hebart H, Bollinger C, Fisch P, Sarfati J, Meisner C, et al (2002) Analysis of T-cell responses to Aspergillus fumigatus antigens in healthy individuals and patients with hematologic malignancies. Blood 100: 4521–8. 12393638 10.1182/blood-2002-01-0265

[3] Perruccio K, Tosti A, Burchielli E, Topini F, Ruggeri L, et al (2005) Transferring functional immune responses to pathogens after haploidentical hematopoietic transplantation. Blood 106: 4397–406. 16123217 10.1182/blood-2005-05-1775 PMC1895249

[4] Chaudhary N, Staab JF, Marr KA (2010) Healthy human T-Cell Responses to Aspergillus fumigatus antigens. PLoS One 5: e9036. 20174463 10.1371/journal.pone.0009036 PMC2822840

[5] Bozza S, Clavaud C, Giovannini G, Fontaine T, Beauvais A, et al (2009) Immune sensing of Aspergillus fumigatus proteins, glycolipids, and polysaccharides and the impact on Th immunity and vaccination. J Immunol 183: 2407–14. 19625642 10.4049/jimmunol.0900961

[6] De Pauw B, Walsh TJ, Donnelly JP, Stevens DA, Edwards JE, et al (2008) Revised definitions of invasive fungal disease from the European Organization for Research and Treatment of Cancer/Invasive Fungal Infections Cooperative Group and the National Institute of Allergy and Infectious Diseases Mycoses Study Group (EORTC/MSG) Consensus Group. Clin Infect Dis 46: 1813–21. 18462102 10.1086/588660 PMC2671227

[7] Potenza L, Vallerini D, Barozzi P, Riva G, Forghieri F, et al (2011) *Mucorales*-specific T cells emerge in the course of invasive mucormycosis and may be used as a surrogate diagnostic marker in high-risk patients. Blood 118: 5416–19. 21931119 10.1182/blood-2011-07-366526

[8] Potenza L, Barozzi P, Vallerini D, Bosco R, Quadrelli C, et al (2007) Diagnosis of invasive aspergillosis by tracking Aspergillus-specific T cells in hematologic patients with pulmonary infiltrates. Leukemia 21: 578–81. 17215858 10.1038/sj.leu.2404504

[9] Riva G, Luppi M, Barozzi P, Quadrelli C, Basso S, et al (2010) Emergence of BCR-ABL-specific cytotoxic T cells in the bone marrow of patients with Ph+ acute lymphoblastic leukemia during long-term imatinib mesylate treatment. Blood 115: 1512–8. 20007806 10.1182/blood-2009-06-230391

[10] Arroyo J, Sarfati J, Baixench MT, Ragni E, Guillén M, et al (2007) The GPI-anchored Gas and Crh families are fungal antigens. Yeast 24: 289–96. 17397107 10.1002/yea.1480

[11] Sarfati J, Monod M, Recco P, Sulahian A, Pinel C, et al (2006) Recombinant antigens as diagnostic markers for aspergillosis. Diagn Microbiol Infect Dis 55: 279–91. 16626916 10.1016/j.diagmicrobio.2006.02.002

[12] Brodoefel H, Vogel M, Hebart H, Einsele H, Vonthein R, et al (2006) Long-term CT follow-up in 40 non-HIV immunocompromised patients with invasive pulmonary aspergillosis: kinetics of CT morphology and correlation with clinical findings and outcome. AJR Am J Roentgenol 187: 404–13. 16861545 10.2214/AJR.05.0513

[13] Caillot D, Couaillier JF, Bernard A, Casasnovas O, Denning DW, et al (2001) Increasing volume and changing characteristics of invasive pulmonary aspergillosis on sequential thoracic computed tomography scans in patients with neutropenia. J Clin Oncol 19: 253–9. 11134220 10.1200/JCO.2001.19.1.253

[14] Cobb BA, Wang Q, Tzianabos AO, Kasper DL (2004) Polysaccharide processing and presentation by the MHCII pathway. Cell 117: 677–687. 15163414 10.016/j.cell.2004.05.001 PMC2917993

[15] Ryan SO, Bonomo JA, Zhao F, Cobb BA (2011) MHCII glycosylation modulates Bacteroides fragilis carbohydrate antigen presentation. J Exp Med 208: 1041–53. 21502329 10.1084/jem.20100508 PMC3092352

[16] Carvalho A, De Luca A, Bozza S, Cunha C, D’Angelo C, et al (2012) TLR3 essentially promotes protective class I-restricted memory CD8+ T-cell responses to Aspergillus fumigatus in hematopoietic transplanted patients. Blood 119: 967–77. 22147891 10.1182/blood-2011-06-362582

[17] Chai LY, van de Veerdonk F, Marijnissen RJ, Cheng SC, Khoo AL, et al (2010) Anti-Aspergillus human host defence relies on type 1 T helper (Th1), rather than type 17 T helper (Th17), cellular immunity. Immunology 130: 46–54. 20002791 10.1111/j.1365-2567.2009.03211.x PMC2855792

[18] Rezvani K, Yong AS, Tawab A, Jafarpour B, Eniafe R, et al (2009) Ex vivo characterization of polyclonal memory CD8 T-cell responses to PRAME-specific peptides in patients with acute lymphoblastic leukemia and acute and chronic myeloid leukemia. Blood 113: 2245–2255. 18988867 10.1182/blood-2008-03-144071 PMC2652370

[19] Karlsson AC, Martin JN, Younger SR, Bredt BM, Epling L, et al (2003) Comparison of the ELISPOT and cytokine flow cytometry assays for the enumeration of antigen-specific T cells. J Immunol Methods 283: 141–153. 14659906 10.1016/j.jim.2003.09.001

[20] Beck O, Topp MS, Koehl U, Roilides E, Simitsopoulou M, et al (2006) Generation of highly purified and functionally active human TH1 cells against Aspergillus fumigatus. Blood 107: 2562–9. 16322466 10.1182/blood-2005-04-1660

[21] Stuehler C, Khanna A, Bozza S, Zelante T, Moretti S, et al (2011) Cross-protective TH1 immunity against *Aspergillus fumigatus* and *Candida albicans* . Blood 117: 5881–91. 21441461 10.1182/blood-2010-12-325084

[22] Ramadan G, Davies B, Kurup VP, Keever-Taylor CA (2005) Generation of cytotoxic T cell responses directed to human leucocyte antigen Class I restricted epitopes from the Aspergillus f16 allergen. Clin Exp Immunol 140: 81–91. 15762878 10.1111/j.1365-2249.2005.02738.x PMC1809331

[23] Ramadan G, Davies B, Kurup VP, Keever-Taylor CA (2005) Generation of Th1 T cell reponses directed to a HLA Class II restricted epitope from *Aspergillus* f16 allergen. Clin Exp Immunol 139: 257–67. 15654824 10.1111/j.1365-2249.2005.02699.x PMC1809287

